# The Capabilities of Boltzmann Machines to Detect and Reconstruct Ising System’s Configurations from a Given Temperature

**DOI:** 10.3390/e25121649

**Published:** 2023-12-12

**Authors:** Mauricio A. Valle

**Affiliations:** Facultad de Economía y Negocios, Universidad Finis Terrae, Santiago 7501015, Chile; mvalle@uft.cl

**Keywords:** restricted Boltzmann machine, Ising model, learning representation, multilayer perceptron, crossover

## Abstract

The restricted Boltzmann machine (RBM) is a generative neural network that can learn in an unsupervised way. This machine has been proven to help understand complex systems, using its ability to generate samples of the system with the same observed distribution. In this work, an Ising system is simulated, creating configurations via Monte Carlo sampling and then using them to train RBMs at different temperatures. Then, 1. the ability of the machine to reconstruct system configurations and 2. its ability to be used as a detector of configurations at specific temperatures are evaluated. The results indicate that the RBM reconstructs configurations following a distribution similar to the original one, but only when the system is in a disordered phase. In an ordered phase, the RBM faces levels of irreproducibility of the configurations in the presence of bimodality, even when the physical observables agree with the theoretical ones. On the other hand, independent of the phase of the system, the information embodied in the neural network weights is sufficient to discriminate whether the configurations come from a given temperature well. The learned representations of the RBM can discriminate system configurations at different temperatures, promising interesting applications in real systems that could help recognize crossover phenomena.

## 1. Introduction

Since Hinton and Salakhutdinov [1] introduced the restricted Boltzmann machines (RBMs), there have been many applications and research in which unsupervised learning using this type of neural network has allowed researchers to find complex representations of the input data in a compressed form in several disciplines. For example, the discovery of patterns of coevolution between amino acids in protein sequences [2], the capture of higher-order statistical dependencies in ECG signals and their reconstruction [3], the creation of representative vectors of speech in speaker recognition [4] and the finding of product cross-categories dependencies obtained from sampling market baskets [5], just to mention a few examples.

To achieve these high-level representations, the RBM must initiate a learning process that consists of an iterative adjustment of the weight’s connections between neurons in the input and the hidden layers, such that the likelihood of the training data being used is maximized. Once this process is completed, the neural network can be used to *generate* or *reconstruct* new samples from the learned probability distribution. In traditional feedforward neural networks, the information flows forward to calculate an error between the input value and the prediction and then adjusts the weights in proportion to that error (the backpropagation error). However, in an RBM, the learning is based on a process called *contrastive divergence* [6], which allows for a more-efficient and faster convergence than other traditional methods such as simulated annealing [7] and sequential Gibbs sampling [8] (for a more exhaustive review of the learning process, please review Zhang and colleagues [9]). This particular distinction makes RBMs a type of neural network with sample-generation and learning-representation capabilities. Thus, the RBM trained with appropriate inputs provides an interesting tool to represent complex dependencies in the data through samples synthetically generated from the neural network itself.

Recent studies have used Boltzmann machines to produce generative samples on the paradigmatic Ising models. For example, the process of generating samples using RBMs accelerates the Monte Carlo simulation of the system, identifying distinct patterns of clusters in the lattice [10]. Similar to this work, an RBM is trained from Metropolis samples of an Ising system at a fixed temperature, and they analyze the ability of the machine to reproduce salient features of phase transition [11,12].

Modeling the thermodynamic observables of many-body physical systems, such as, for example, that of an Ising system through unsupervised learning by using Boltzmann machines, has captured the attention of researchers who have found interesting results. First, the trained Boltzmann machines are able to generate spin states that capture thermodynamic observables (i.e., energy, magnetization, specific heat and susceptibility) similar to the original ones generated by Monte Carlo simulation methods [13,14], even identifying phase transitions [15,16]. Second, the appearance of the *RBM flow*, a phenomenon consisting of the convergence of the machine to a fixed point (close to the critical temperature) after iterative reconstructions of spin configurations [16,17,18,19]. Third, the possibility of characterizing the Ising phase transition from the matrix of weights connecting the visible and hidden units of the RBM [14,18,20].

There is evidence that RBMs are able to capture the distribution of the Ising model [21,22] and also detect phase transitions without external help from a human [23]; however, the present work does not deal with the latter aspect but rather with the RBM’s ability to detect input configurations that do or do not correspond to a given system temperature even when the RBM has difficulty generating samples that are physically incompatible with the Ising system.

This study has two primary purposes: The first is to show that the RBM possesses some synthetic-sample-generation problems, particularly in situations where the distribution is bimodal, such as in an Ising system when the system temperature is below the critical temperature. Second, despite this difficulty, the trained RBM encodes sufficient information (with only one hidden layer) through the network weights to successfully guess the temperature of a system configuration.

The following sections of the manuscript are organized as follows: I describe the problem of RBMs to generate samples under certain conditions. I then describe the methodology used to train the RBM and generate Ising samples. Next, I describe the ability of the RBM to generate representations of various sample configurations and to discriminate whether or not the samples correspond to a certain temperature of the system. Finally, I show how these results can be helpful and applied in contexts other than an Ising system.

## 2. Materials and Methods

This work’s development involves four phases, which can be described as: 1. Monte Carlo simulations of the Ising system at a specific temperature, 2. training of the RBM, 3. training a multilayer perceptron (MPL) and 4. evaluation of the MLP. Most of the methods used in this work are standard and well known, so I will briefly describe the Monte Carlo generation of Ising samples and the RBM training, with references in case the reader needs to go deeper into the algorithms.

### 2.1. Ising Simulations

To keep the analysis computationally simple, we use a two-dimensional lattice as the object of study of size L×L, with periodic border conditions, i.e., borders wrapped around the opposite site, being L=10, i.e., N=100 spins, which can adopt values $s_{i}=\pm 1$, i=1,…,N. The couplings are between spin *i* and *j*, and $J_{ij}$ is ferromagnetic with $J_{ij}=J=1$ and external magnetic field h=0. In this case, the Hamiltonian of the system for a particular state s = $\left(s_{1},s_{2},\ldots ,s_{N}\right)$ can be described as

(1) $$H\left(s\right)=-J\sum_{\langle i,j\rangle }s_{i}s_{j}$$

where 〈i,j〉 represents the nearest neighbors for spin *i* and *j*. For any given spin, the number of nearest spins will be always another four.

**Figure 1:**
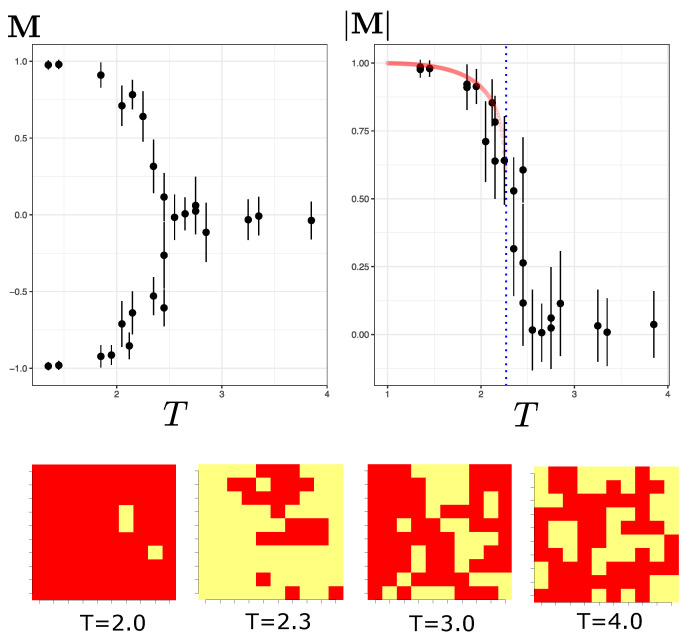
The left part of the figure shows the shape of the magnetization curve of the spins as a function of temperature (the M-*T* plane). The right part is the absolute value of the magnetization |M|. The dotted blue line represents the critical temperature for this system ($T_{c}\approx 2.269$). The red-colored curve is the theoretical approximation corresponding to the Onsager’s solution [25] for $T<T_{c}$, in which $\left|M\right|={\left(1-{\sin h}^{-4}\left(2J\right)\right)}^{1/8}$. The four figures below represent sample configurations of the Ising system for L=10 at different temperatures.

The Metropolis Monte Carlo (MC) method [24] is used to create system configurations at a given temperature *T*. I follow the procedure similar to the one in Iso, Shiba, Yokoo (2018) [17] and Morningstar and Melko (2018) [12], in which I prepare $2^{14}$ MC simulated configurations from the target probability distribution of a configuration s given by $p\left(s,T\right)=1/Z_{s}e^{-H(s)/T}$. To achieve this, the system must be in thermal equilibrium at temperature *T*. By observing the magnetization *M* and energy *E* per site during the Metropolis sweeps process (see Equation (2)) outside the critical region, the equilibrium state tends to be achieved quickly in the first few iterations. For calculating observables (such as those indicated in Figure 1), I discard the first 8000 configurations and then select the remaining configurations but spaced every 100 successive configurations so that they are less correlated.

Initially, the system starts with a random s configuration, and then the orientation of each spin is flipped according to

(2) $$p_{i}=\left\{\begin{array}{cc} 1 & \;\mathrm{if}\;\Delta H_{i}<0\\ e^{-\Delta H_{i}/T} & \;\mathrm{if}\;\Delta H_{i}\geq 0 \end{array}\right.$$

where $\Delta H_{i}$ is the energy change in the system when we change the spin orientation *i*. After many iterations of flipping all the spins, the configuration begins to converge to an equilibrium state at temperature *T*. In the simulations, I flipped each of the *N* spins in random order. This represents a Metropolis sweep.

It should be noted that at a temperature near the critical temperature (e.g., at T=2.3), the critical slowing down manifests itself, making it more challenging to obtain steady-state configurations. A visual inspection of *M* and *E* after many iterations (over 5000) reveals that the system achieves some but not complete stabilization due to persistent low-magnitude oscillations. By selecting samples at this temperature, a higher error level in the observables manifests itself, which is visible in the error bars in Figure 1.

To obtain an idea of the average orientation or magnetization, $M=\frac{1}{N}\sum s_{i}$ of the system configurations, Figure 1 shows the typical magnetization curve in a temperature range from 1.3 to 3.7. In this range of temperature, we can see the magnetization of the system below and above the critical system temperature $T_{c}$ ($T_{c}\approx 2.269J$ for a 2D lattice [25]).

A visible phase transition can be seen in Figure 1 between T=2 and T=2.5. It is important to note that for a temperature lower than $T_{c}$, the average orientation of the spins tends to follow one of the two ground states (almost all spins up or down). The RBM should learn that both equilibrium configurations can be attributed at the same temperature. At temperatures above the critical value, spins tend to be randomly distributed. Thus, the results of all these configurations for each temperature will be the input data used to train the Boltzmann machine.

### 2.2. Restricted Boltzmann Machine Learning

In this section, I review the basic algorithm for training RBMs. For more technical details of the Boltzmann machine learning process, the reader can review [26,27,28].

In its simplest form, the RBM consists of two layers: an input layer with m=nV, $v=(v_{1},\ldots ,v_{m})$ visible units and an invisible layer with n=nH, $h=(h_{1},\ldots ,h_{n})$ hidden units. It is equivalent to a bipartite network in which the connections are between the neurons of the input layer and the hidden layer, but connections between neurons of the same layer are not allowed. It is in the hidden layer where the machine extracts the statistical features of the input data.

For training purposes, the input to the visible layer is the Ising configurations sampled with MC. The Ising system has $N=L^{2}=100$ spins, and then the number of visible units of the machine will be nV=m=100.

Regarding the number of hidden units, at present, such a choice seems to be of empirical matter. Too high a number (e.g., nH>nV) seems to be unnecessary since a high percentage of neurons in this layer become inactive and encode very little information from the input [29], and the network tends to learn too many noisy fluctuations [17]. On the other hand, with a very low number of units, there is a risk of limiting learning and failing to recognize complex patterns and interrelationships between variables in the input units [2,26]. The number of hidden units that seems to work well in practice is close to half the number of visible units, so nH=n=64.

The main objective of the RBM can be understood as a neural network that adjusts its weights (connections between the visible and hidden units) such that the learned p(v) distribution models the underlying q(v) distribution in the training data. The above is equivalent to maximizing the likelihood function given by:

(3) $$\ln L\left(\theta |S\right)=\sum_{i=1}^{l}\ln p\left(v_{i}|\theta \right).$$

where S is the given dataset and θ={W,B,C} is the set of RBM parameters. Expressed differently, RBM learning is an optimization process that consists of minimizing the distance between q(v) and p(v), or the Kullback–Leibler (KL) divergence:

(4) $$\mathrm{KL}\left(p||q\right)=\sum_{v\in \omega }p\left(v\right)\ln\frac{p(v)}{q(v)}=\sum_{v\in \omega }p\left(v\right)\ln p\left(v\right)-\sum_{v\in \omega }p\left(v\right)\ln q\left(v\right)$$

The KL divergence is minimized by adjusting the weights of the network W connecting each visible unit to all hidden units and its biases B and C for visible and hidden units, respectively. The description of the p(v) distribution of learning from the joint probability distribution of (v,h) will be given by

(5) $$p\left(v\right)=\sum_{h}p\left(v,h\right)=\frac{1}{Z}\sum_{h}\exp\left(-E\left(v,h\right)\right)$$

where $Z=\sum_{v,h}\exp\left(-E\left(v,h\right)\right)$ is the partition function with its energy function:

(6) $$E\left(v,h\right)=-\sum_{i=1}^{m}\sum_{j=1}^{n}w_{ij}h_{i}v_{j}-\sum_{j=1}^{m}b_{j}v_{j}-\sum_{i=1}^{n}c_{i}h_{i}$$

Approximating the expectation over *q* in Equation (4) with training samples from *q* results in the log-likelihood function Equation (3), so maximizing the log likelihood is the same as minimizing the KL divergence.

In RBMs, the gradient of the log likelihood can be written in terms of the sum of two expectations as

(7) $$\frac{\partial \ln L(\theta |v)}{\partial w_{ij}}\propto {\langle v_{i}h_{i}\rangle }_{\mathrm{data}}-{\langle v_{i}h_{i}\rangle }_{\mathrm{model}}$$

where the notation 〈…〉 denotes expectations. A similar expression for the log likelihood with respect to the bias parameters of visible $b_{j}$ and hidden units $c_{i}$ is used. The problem is that the second expectation ${\langle v_{i}h_{j}\rangle }_{\mathrm{model}}$ is difficult to obtain since it requires using enough MC sampling, which makes the process too slow. Instead, it has been found that obtaining estimates of this expectation can be performed through Gibbs sampling chain running for *k* steps (usually works well with k=1), a process called *contrastive divergence* [6,30]. Thus, the gradients in the direction of each parameter are obtained by estimating the expectations on p(v) in Equation (7) in sample batches $v^{k}$. Finally, the upgrading of the network parameters θ is made iteratively such that

(8) $$\begin{array}{cc} \Delta w_{ij} & =\eta ({\langle v_{i}h_{j}\rangle }_{\mathrm{data}}-{\langle v_{i}^{k}h_{j}\rangle }_{\mathrm{model}})\\ \Delta b_{i} & =\eta ({\langle v_{i}\rangle }_{\mathrm{data}}-{\langle v_{i}^{k}\rangle }_{\mathrm{model}})\\ \Delta c_{j} & =\eta ({\langle h_{j}\rangle }_{\mathrm{data}}-{\langle h_{j}\rangle }_{\mathrm{model}}) \end{array}$$

where η is the learning rate. For RBM training using samples from the Ising system at a specific temperature, the initial learning rate is 0.0001 and then progressively decreases across epochs with a decay of 0.01 with a momentum of 0.8. The number of epochs is usually between 200 and 500 and depends on the convergence of the reconstruction error (the mean square deviation between the original and reconstructed data and an increasing pseudolikelihood). The Gibbs sampling number in the negative training phase is k=20. The size of the batch size presented to the RBM on each epoch was 128 configurations. The initial values of the θ parameters were initialized with random values drawn from a Gaussian distribution with a zero mean and standard deviation of 0.01 [26]. During learning, batches of system configurations of the Ising system at different temperatures of size 100 are presented to the input layer. For the convenience of the RBM calculations, the original values of the spins in state $z_{i}$, +1 or −1, are rescaled to values of +1 or 0, respectively, using $s_{i}=0.5\left(z_{i}+1\right)$.

## 3. Simulation Results

Before beginning the analysis of training and classification, I present a simple example to denote the problem of the RBM to generate new samples. This problem manifests itself when the original distribution presents a bimodal distribution of the magnetizations of spins.

### 3.1. Reconstruction under Bimodal Spins Distribution

**Figure 2:**
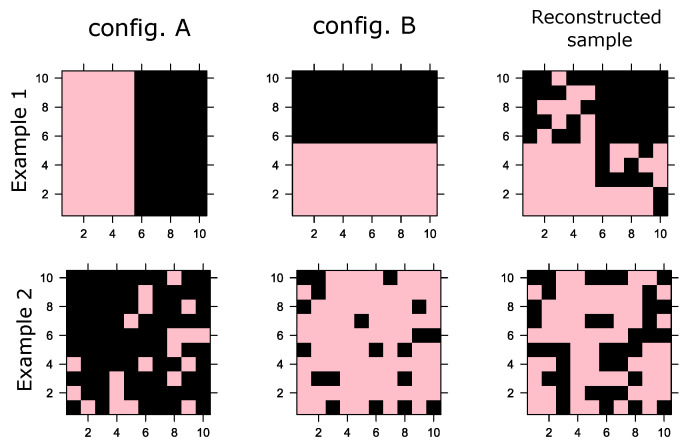
Two examples in which two RBMs are trained with only two configurations (**A**,**B**). The third column shows a typical reconstruction achieved by the RBM. For both cases, the training configurations have half spins at +1 and half at −1, i.e., M=0. The reconstruction also has M=0.5; however, the physical arrangement of each spin’s orientations is incorrect.

Let us assume two fictitious configurations: A and B, as shown in Figure 2. Let us define the magnetization of a configuration s as $M=1/N\sum_{i}^{N}s_{i}$. In Example 1, M=0 for both configurations; i.e., half of the spins are in −1 state and the rest are in +1 states. Consequently, the magnetization distribution of the samples is conserved at zero, but, especially, both configurations have marked concentration spins at −1 and +1 at different locations, as can be seen in Example 1. In contrast, in Example 2, the one training configuration has M=−0.8 (predominantly in the −1 state) and the other has M=0.8 (predominantly in the +1 state), a distinctive bimodal distribution of spin magnetization; however, the mean of the magnetization distribution of the training data is 0.

As shown in Example 1, the reconstructed sample (using k=20 iterations of Gibbs samplings) results in a configuration that complies with the mean orientation of the spins (M=0). However, it reconstructs a sample that violates the spatial correlations between the spins. In Example 2, the same thing happens, reconstructing a sample in which also M=0 but does not physically comply with a configuration predominantly with spins at −1 or +1. This problem is not in itself a machine failure since Gibbs sampling is essentially a stochastic procedure compliant with generating samples that satisfy the training configurations on average.

These examples are only intended to show that although the RBM manages to generate synthetic samples that comply with the above when observing the orientation of the spins, they do not correctly reproduce the spatial distribution of the orientations; i.e., they are configurations of the system that are physically not supported and fail to capture the large clusters present in the examples.

In the next section, I show that even though the machine cannot generate physically correct samples, it can still correctly store information on the temperature from which the training samples were generated.

### 3.2. Revealing RBM’s Representations of Spin Configurations

This section presents the training results of an RBM and analyzes its ability to generate Ising coherent samples. I then analyze the learned representations of the machine, projecting the values of the hidden units into a 2D plane.

For the 2D Ising system with N = 100 spins, I trained an RBM at a single temperature at T=2.3 (near the critical temperature of $T_{c}=2.269$) by using M=2048 configurations generated from the MC sampling at that temperature. The number of hidden units is nH=64. The hyperparameters used are described in Section 2.2. Let us call this trained machine ${\mathrm{RBM}}_{T=2.3}$ to denote that it is a restricted Boltzmann machine trained with configurations at a temperature of T=2.3.

It is interesting to evaluate the ability of the RBM to learn representations between configurations of the same temperature at which the RBM was trained from other configurations generated at other temperatures. This approach is different from what has been conducted before, in which some kind of feedforward neural network is trained to determine the temperature of a configuration [21]. The idea here is to analyze the ability of the RBM to detect configurations at a given temperature. A total of 8192 different configurations were presented to the ${\mathrm{RBM}}_{T=2.3}$, 1024 for each temperature set T={2.0,2.2,2.3,2.4,2.5,2.7}; two temperatures for the ordered phase, one near criticality (where magnetization converges to zero) and three for the disordered phase. Then, the resulting activation probabilities of the hidden units are projected on a 2D plane by using the first two principal components. The activation probabilities of the hidden units are computed by using Equation (A2) (see Appendix A).

**Figure 3:**
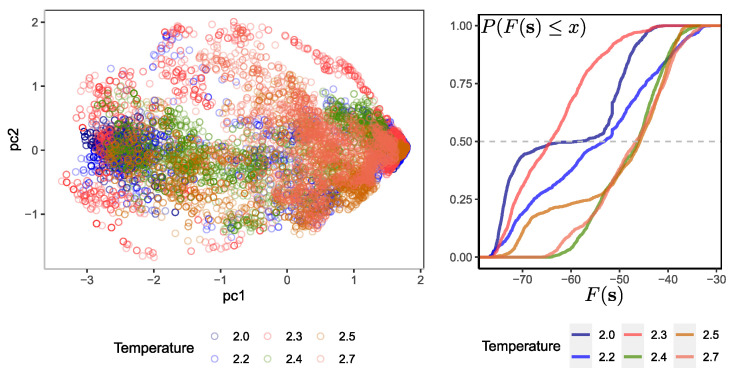
(**Left**) Two principal components plot of the hidden values’ probabilities activation for a sample of configurations at different temperatures. The total variance explained by these two components is 87.5%. (**Right**) The empirical cumulative distribution function for the free energy F(s). The gray dashed horizontal line denotes the P(F≤x)=0.5. Note: the input vectors fed to the ${\mathrm{RBM}}_{T=2.3}$ are samples different from those used for training.

Similar to what was found by [15], the variation along the first component is stronger than in the second. However, projecting the probabilities of the hidden RBM units provides a different perspective than doing the same directly on the original configurations. Figure 3 on the left shows that the configurations at T=2.3 tend to lie in the plane with a larger spread in both components than other samples at different temperatures. This denotes long fluctuations in the system’s dynamics and the effect of long-range spin ordering. On the other hand, it is observed in the projection that the components are concentrated at the opposite poles of the first component at low temperatures, and those at higher temperatures are scattered in a thinner band along the first component. These characteristics can help discriminate between configurations coming from the system at a near-critical temperature and other ones.

For a specific input configuration s, the log probability that the ${\mathrm{RBM}}_{T}$ assigns to a specific input vector s is equivalent to the likelihood that this configuration belongs to the temperature *T*, which can be computed as

(9) $$\log p\left(s|T\right)=-F\left(s\right)-\log Z_{T},$$

where $Z_{T}$ is the partition function of the ${\mathrm{RBM}}_{T}$ and F(s) is the free energy computed as

(10) $$F\left(s\right)=-\sum_{j}b_{j}s_{j}-\sum_{i}\log\left(1+e^{x_{i}}\right),$$

where $x_{i}=c_{i}+\sum_{j}w_{ij}s_{j}$. The partition function $Z_{T}$ can be considered here as a constant so that logp(s|T) is proportional to free energy.

The idea is to observe and compare the log likelihood distribution of p(s|T) via the free energy (Equation (9)) for configurations at different temperatures over an RBM trained for a specific temperature $T_{i}$. By calculating the distribution of F(s) over configurations at $T_{i}$ and other different temperatures, say $T_{j}$, i≠j, then one should expect that the log likelihood of those configurations at $T_{i}$ should be larger than those at a temperature $T_{j}$. In this way, one can observe the machine’s discrimination potential to differentiate configurations at different temperatures. This idea has been used to use RBMs as classifiers in other fields, such as spectral classification [31].

Figure 3 on the right shows the cumulative distribution probability of the free energy calculated over several configurations at different temperatures. Recall that the set of parameters θ of the RBM is always the same and corresponds to the trained RBM at T=2.3. As expected, those input vectors coming from a temperature equal to that of the RBM tend to have lower free energy than other configurations coming from a temperature T≠2.3. In fact, it can be seen from the samples used that there are configurations at T=2.0 that possess a slightly lower free energy than configurations at T=2.3. This could be a source of confusion in the ability of the machine to discriminate.

### 3.3. Sampling Configurations from the ${\mathrm{RBM}}_{T=2.3}$

As indicated in Section 2.2, the trained RBM can approximate the data distribution with samples from v∼*q* through a *p* learned distribution. This approximation is conducted via the generative model such that the distribution *p* remains a function of the machine parameters θ. Once trained, the RBM is used as a generative model of p(θ) to generate new configurations using Equations (A2) and (A3) (see Appendix A) through the block Gibbs sampling procedure: from an initial random spins system configuration $v_{0}$, $p(h_{0}|v_{0})$ is computed, from which $h_{0}$ is obtained. Then, $p(v_{1}|h_{0})$ is computed and the sample $v_{1}$ is obtained. We repeat this process of updating for visible and hidden units *k* times to obtain a distribution q(θ).

For the purposes of this study, with k=20 repetitions of Gibbs samplings (increasing *k* does not change the results), it is possible to obtain a sample of configurations with a distribution *q* similar to the original *p* used to train the RBM. Using this procedure, I generated 2048 synthetic configurations.

For clarity in the comparison between the configurations sampled by MCMC and generated by the RBM, the following observables are computed: First, $\langle s_{i}\rangle$ represents the mean of the *i*th spin orientation of the lattice computed from the configurations sampled by MC and generated by the RBM. Second, the pairwise products between spins, $\langle s_{i}s_{j}\rangle =\frac{1}{N}\sum_{\forall i\neq j}s_{i}s_{j}$, are the average of the multiplications between each pair of spins using all the sampled configurations. Third, the magnetization $M=\frac{1}{N}\sum_{i}^{N}s_{i}$ is the average of the states of each spin of a given configuration. Finally, the energy density of the system is $E=-\frac{1}{N}\sum_{\langle ij\rangle }s_{i}s_{j}$ for a given configuration, with 〈ij〉 being the nearest neighbors per spin for *i* and *j*.

**Figure 4:**
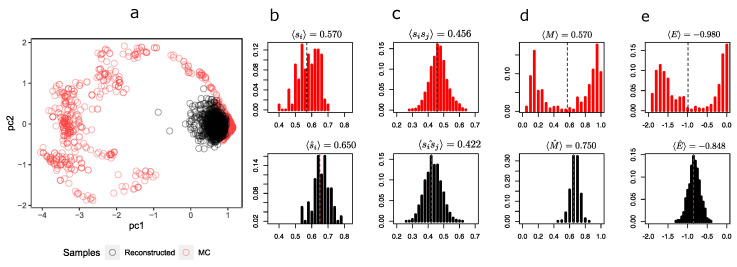
(**a**) First two principal components of 2048 reconstructed samples via Gibbs samplings using k=10 steps with the ${\mathrm{RBM}}_{T=2.3}$ and 2048 MC samples at T=2.3. The explained variance with these two components was 90.3%. (**b**) Distribution of mean orientations of every spin of the lattice. (**c**) Distribution of pairwise product means between spins. (**d**) Distribution of the magnetizations of sample configurations of the Ising system. (**e**) Distribution of the system energies. Note: For all plots, black represents data from reconstructed samples and red represents data from MC samples.

To compare the representations that the RBM sees in the hidden layer, Figure 4 shows a scatterplot of the first two components of the hidden unit values of the MC samples and the synthetic configurations.

It is possible to observe from Figure 4a that the synthetic configurations (in black) tend to be grouped in the same place, being under-represented in relation to the greater heterogeneity of MC’s sample configurations (in red). The distribution of the magnetizations (Figure 4d in black) simulated by the RBM fails to capture the bimodality produced by symmetry breaking in these two predominant states.

At this temperature, the system has configurations with both negative (in the figure with M<0.5) and positive (M>0.5) magnetizations, while the reconstructions are all with a magnetization close to 〈M〉=0.750. Notwithstanding the above, the RBM does a decent job of recovering the average orientations of the spins $\langle s_{i}\rangle$ and pairwise products $\langle s_{i}s_{j}\rangle$ in Figure 4a and Figure 4b, respectively. This is expected because RBM training is essentially based on maximizing the p(v,h) log likelihood, i.e., finding a distribution p(v) that models the underlying distribution q(v) as indicated in Equation (5), which necessarily implies achieving consistency between the first moment and second moment of the distribution of q(v) and consequently also with the pairwise products $\langle s_{i}s_{j}\rangle$. In Figure 4e, it is also observed that the energy distribution of MC and synthetic configurations only agree on the mean (at least they are very similar); however, both distributions differ in their shapes. A similar situation occurs at T=2.2 (see Appendix C.1), in which the mean orientation of the spins is correctly recovered but the mean pairwise product clearly starts to differ, revealing a problem with the synthetic configurations. At T=3.0 (see Appendix C.2), the RBM correctly recovers the observables and distributions.

A manifestation of the learning problem with bimodal distributions is also observed in the error reconstruction of the configurations, particularly at low temperatures, which is more severe. The difference in the evolution of these errors in the learning process of an RBM with ordered and disordered phase configurations can be observed in Appendix B.

It would be important to note at this point that the RBM does not have the inability to reproduce the statistics at different temperatures of the Ising system. The means of observables, such as the mean orientation of the spins and the pairwise product between spins, are quite similar between those of the data and those of the model. However, particularly at very low temperatures, when the system predominantly has states on −1 or +1 spins, the distribution of the magnetizations simulated by the RBM fails to capture the bimodality produced by symmetry breaking in these two predominant states.

### 3.4. Additional Training

The RBM does not entirely fail to reproduce specific statistics (the mean orientation and pairwise spin products) about those coming from the Ising system, a matter that other studies have shown that the RBM can perform quite well. What it fails to reproduce correctly, particularly at low temperatures, are the system configurations, in which at temperatures below the critical temperature of the system, the spins are highly correlated with large clusters with the same polarization. Specifically, it is observed that under these conditions, the magnetization *M* and the system’s energy do not agree with the real ones. The RBM does not seem to capture the physical connections between the spins in the 2D Ising lattice. A situation similar to this one has also been reported by Azizi and Pleimling (2021) [32].

Given the clustered nature of the distribution of Ising-system configurations at low temperatures, it is possible that the Gibbs sampling process to estimate the negative part of the log-likelihood gradient during training (Equation (7)) fails to reach an equilibrium state; consequently, the RBM samples out-of-equilibrium configurations [33], resulting in biased configurations. To analyze this issue, additional training was carried out with longer MCMC steps and also using Persistent Contrastive Divergence (PCD) [34]. PCD can be considered as an improvement over contrastive divergence (CD), in which the final configurations of each Markov chain are used as a starting point in the next chain. Decelle and coauthors [33] showed that the CD method is often poor because the sampling of the Markov chains in equilibrium differs from the training dataset’s distribution. In this sense, PCD could provide better results.

**Figure 5:**
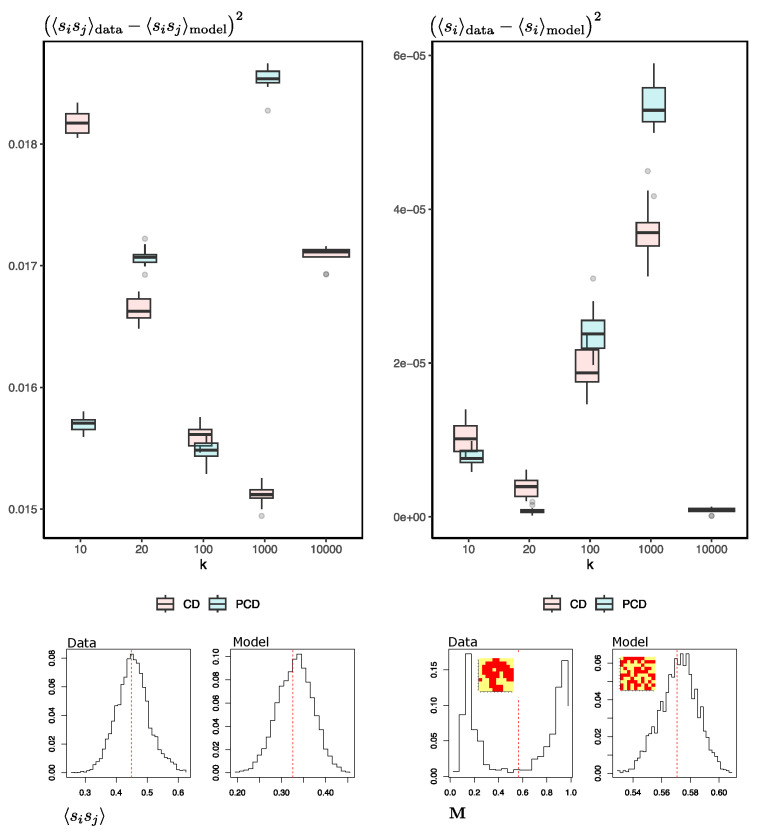
Comparison of real Ising-system samples with synthetic samples generated by RBMs with different numbers of MCMC Gibbs samplings *k*, using contrastive divergence (CD) and Persistent Contrastive Divergence (PCD). The plots in the lower part of the figure show an example of the distributions of pairwise connections between spins and mean orientations for Ising-system configurations and RBM-generated configurations. A mini-representation of the Ising system, coloring the spins in red-yellow according to their orientation, is found in the Inset of the magnetization distributions to denote the difference between a real system configuration and a synthetic one. All training has been carried out with configurations at T=2.3. The reported boxplots correspond to 15 different trainings.

I chose to conduct the simulations at a temperature T=2.3 close to the critical temperature. At this temperature, we already have evidence of symmetry breaking, where the system tends to form large clusters of neighboring spins with the same orientation. At lower temperatures, this phenomenon is more exacerbated, and the set of spins of the system is represented by a majority in one of the two possible equilibrium states, giving rise to magnetization distributions with a clear bimodality (M≈−1 and M≈+1). Figure 5 shows the degree of fit achieved by the RBM in reproducing synthetic samples by using the squared difference between the mean orientation of the spins of the (test) data and the samples generated by the RBM $\langle s_{i}\rangle$ and likewise for the pairwise products between spins $\langle s_{i}s_{j}\rangle$.

First, the differences between the test data and those generated by the machine do not decrease as we increase the number of MCMC steps. In fact, for the pairwise products, it seems to increase, being minimal at *k* = 10,000. It is worth noting that the averages of the orientation of the reproduced spins fit reasonably well; however, looking at the distribution of the magnetizations of the real and synthetic spins (right-lower part of the Figure, also similar to what happens in Figure 4d), we see that the machine-generated configurations are oblivious to the real ones. Second, this situation does not improve when using PCD. Either way, the dominance of metastable states or clustered data causes the mixing time to increase rapidly during training [35]. This could explain why the RBM-generating configurations do not represent the natural Ising system.

Several other simulations were carried out to evaluate the error of the configurations generated by the RBM at different temperatures and with different levels of randomness or noise in the initial configurations for synthetic-sample generation. Given an s configuration of the Ising system at temperature *T*, let us define the parameter *f*, which is simply the ratio between the number of spin orientations from the MCMC data and the total number of spins of the system (in our case it is 100). So, for example, we can generate a new configuration with f=0.5, which means that half of the spins are random and the other half are the actual orientations of s. If f=0, it is simply a completely random configuration. For a given temperature, we used the RBM to generate synthetic configurations, starting with configurations with different noise values (*f*), and then compared the error with the actual configurations. The error was evaluated as the squared difference of the magnetization of the synthetic and real samples and the squared difference of the pairwise products between spins $\langle s_{i}s_{j}\rangle$.

**Figure 6:**
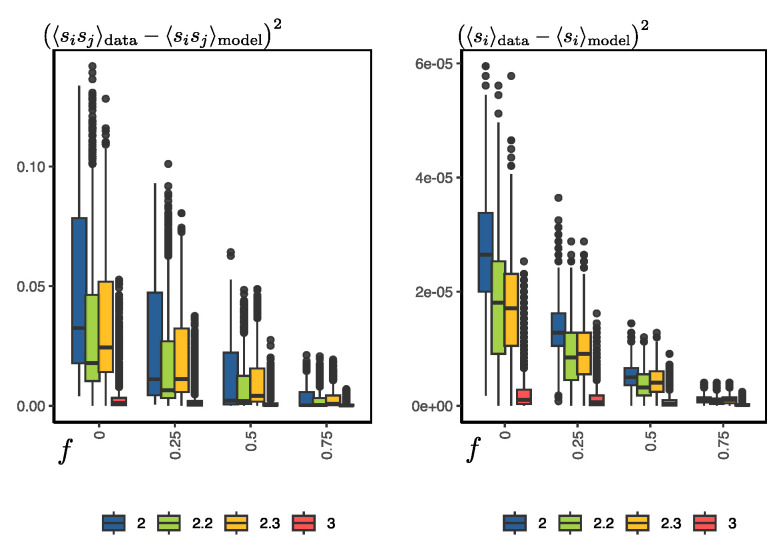
For each temperature T={2.0,2.2,2.3,3.0}, 2048 MCMC-sampled Ising-system configurations (see Section 2.1) not used in RBM learning were taken. For each of these configurations, the noise level *f* is varied.

In Figure 6, we can observe the general results of the simulations. First, the error increases as we increase the level of randomness of the configurations to start the generation process. For example, when we leave 75% of the orientations untouched, the error in the magnetization and pairwise products is close to zero. However, the real test for the RBM is when f=0; i.e., we start the process from totally random configurations. In this case, the error is slightly larger, although it is worth noting that this error does not seem to decrease by increasing the number of Gibbs samplings in the training process or using PCD, as discussed previously (see Figure 5). Second, for temperature T=3, the errors are very close to zero, but as we decrease the temperature, the average error increases slightly, but with a significant increase in the variance. Here, it is worth mentioning that the average magnetization and pairwise error at f=0 and T=2.0 are 15% and 1.7%, respectively. Hence, the main problem lies in the bimodality of the distribution of configurations at temperatures below the critical temperature, which becomes more pronounced at lower temperatures.

**Figure 7:**
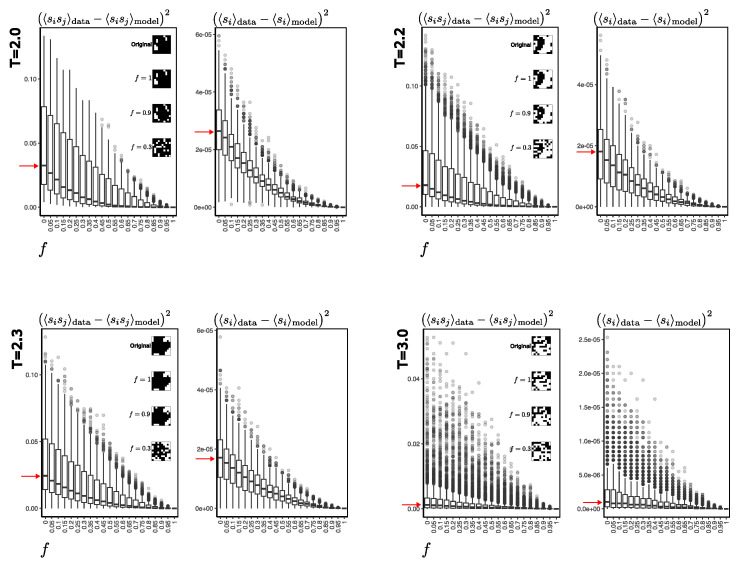
To construct these boxplots, 2048 configurations of the Ising system sampled by MCMC (see Section 2.1) were used for the indicated temperatures. For each of these configurations, a randomization of the orientation of the spins was applied according to the value of the *f* parameter. The resulting configurations are then used as a starting point for generating synthetic samples, which are compared with the original ones to calculate the error. An example of a real configuration and its respective recoveries with different noise levels is shown on the right side of the magnetization error plots to obtain a visual idea of the real and synthetic Ising-system configurations. The red arrow indicates the average error obtained when starting the generation process with totally randomized spins.

A more detailed comparison can be seen in Figure 7. The errors are quite low at higher temperatures (here at T=3.0) compared to lower temperatures, even with noise parameters *f* close to one. For example, at lower temperatures (here at T=2.0 and T=2.2), the RBM has more difficulty recovering the original configurations. From the physical point of view, as the temperature is low, the system is predominantly in two equilibrium states: one where almost all the spins are at +1, and another where almost all are oriented at −1. This condition occurs predominantly at temperatures below the critical temperature, in which there is an increase in the error in the reconstruction of the configurations. Note that, as expected, with low noise values, the RBM will recover the real configurations virtually perfectly.

### 3.5. Training a Critical Spin Configuration’s Detector

Although the RBM is among the types of machines trained under unsupervised learning, its feature representation can help discriminate system configurations that belong to a particular condition. In this case, to verify the ability of the representation achieved by the RBM with the Ising system, I train a multilayer perceptron (MLP) as a binary classifier whose input information is the representation of the RBM in the hidden layer, say p(h|s), and the output *y* of the neural model is a classification indicating whether the configuration s belongs to the system at a temperature *T*.

For MLP training and testing purposes, I generated ten different sample sets of MC configurations of an Ising system as described in Section 2.1. Each set has 16,384 different configurations. About 80% of them are used for training, and the remaining 20% are used for testing. For each set, I generated a class variable *y* indicating 1 if the probability vector p(h|s) belongs to a configuration s at a temperature of T=2.3 or 0 if it belongs to a configuration of any other different temperature. Likewise, to ensure a balanced sampling of classes, for each set, half of the samples correspond to configurations at T=2.3, and the other half correspond to different temperatures of 2.0, 2.2, 2.4, 2.5, 2.7, 3.0 and 3.4. Each training set was used to train 10 different MLPs independently. All MLPs have an input layer with n=64 units, an intermediate layer with two neurons with a RELU activation function and an output neuron with a sigmoid activation function. Initial random weights for the MLP training were set to 0.5. The parameter for weight decay was set to 0.004, and the maximum number of iterations was set to 200.

Table 1 shows the performance of the MLP classifiers. These results appear to be respectable, considering the overlap between the classes and the nature of the problem we are dealing with. This indicates that the hidden layer of the RBM carries enough system information to discriminate whether the configurations in the input layer belong to valid configurations at the system temperature. This is attractive because if we are interested only in some particular system temperature (in this case, the near-critical temperature), training a series of other RBMs at different temperatures is unnecessary to discriminate between other system states. In Appendix D, I show the results of repeating precisely the same MLP training exercise but using a different number of hidden RBM units. In short, it can be seen that there is a marginal impact on the classifier’s ability to discriminate configurations. The higher the number of hidden units, the slightly better the performance of the classifier.

**Figure 8:**
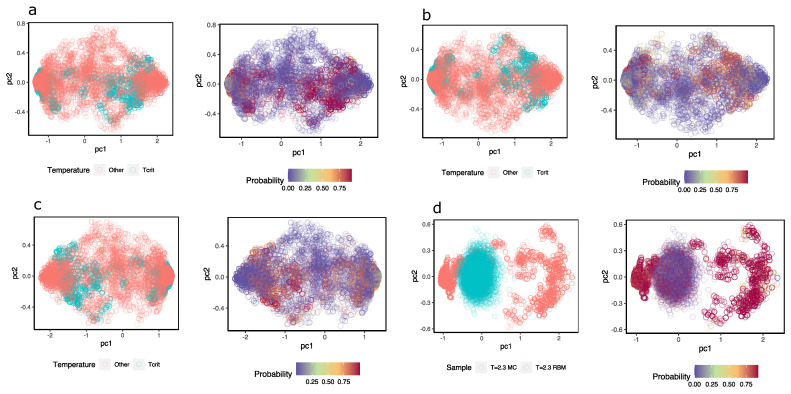
(**a**–**c**) Three exercises of configuration detection at temperature T=2.3. The figures show the first two components of the hidden values calculated with ${\mathrm{RBM}}_{T=2.3}$ in two versions: The first is colored according to the temperature of the configurations; in magenta are configurations at T=2.3 and in pink are configurations at T≠2.3. The second is colored according to the probability that y=1 is what the MLP classifier assigns to each hidden value. (**d**) The first two principal components of configurations at T=2.3; in magenta color configurations reconstructed with Gibbs samplings using k=10 iterations, in pink color configurations from Ising model samples. On the right is the same, but they are colored by probability that the hidden units come from configurations at T=2.3.

To obtain an idea of how the MLP discriminates near-critical temperature configurations from others, Figure 8a–c shows a representation of the hidden units colored according to the class they belong to; next to them, the hidden units are the same, but they are colored according to the probability that the MLP assigns configuration at T=2.3. In this case, different samples were taken from configurations at temperature T=2.3 (magenta) and the same number of samples from configurations with T≠2.3 (in pink). Although a high degree of overlap between the two sets of configurations can be observed, the MLP (three different ones) assign high probabilities to values of the hidden units of configurations at T=2.3 and low probabilities to those that are not.

Additionally, the same idea is shown in Figure 8d but with samples of configurations at T=2.3 and others that the RBM generated. When feeding the hidden unit values of these configurations to the MLP, the MLP successfully recognizes the configurations at T=2.3; however, the reconstructions achieved with the same RBM are classified as configurations other than T=2.3, when in fact, they should not be. This again highlights the problems of the RBM in generating appropriate configurations.

As a complement, in Appendix C, I repeat the same exercise for two other system temperatures—one at a disordered phase temperature at T=3.0 and one at an ordered phase at T=2.2. In the first case, it is possible to observe that the results of generating configurations using ${\mathrm{RBM}}_{T=3.0}$ are much better than those when using ${\mathrm{RBM}}_{T=2.3}$ and also in the second case with ${\mathrm{RBM}}_{T=2.2}$. This is not surprising: with disordered or high-temperature configurations, there are no bimodal distributions, while in the opposite case, as we have already indicated above, with bimodal distributions, the RBM has difficulties generating correct configurations. Despite this problem, the RBMs still encode in the hidden units the information necessary to identify whether or not the configurations belong to the RBM temperature when we use the hidden units as inputs to train MLP classifiers.

Previous research [15,36] has shown that other much simpler unsupervised learning techniques, such as principal component analysis (PCA), can successfully recognize the phase changes in an Ising system. Thus, it can be assumed that it could be a good competitor to RBMs for detecting the temperature of configurations. Additional temperature-detection models of configurations of an Ising system have been added, but only using the PCA components applied directly on training setups. This allows for a comparison of both PCA and RBM performance (under the same MLP architectures described before) in identifying samples of different temperatures.

**Figure 9:**
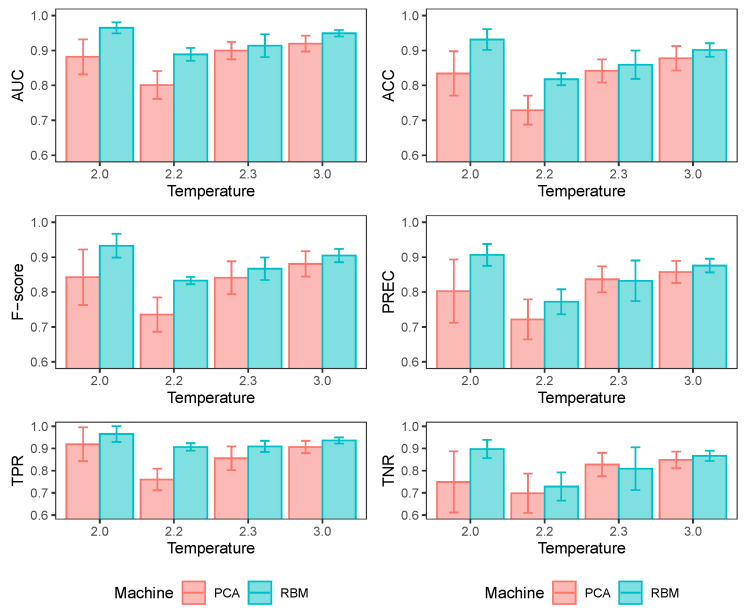
Different performance measurements of temperature detectors trained at different temperatures using MLPs with the same architecture: 64 units in the input layer, an intermediate layer with two units with RELU activation function and an output neuron with sigmoid activation function. The input-layer values differ: for PCA, they are the first 64 components of the training-input configurations; for the RBM, they are the probability vectors p(h|s). I train ten different models in each case and report the averages of the performance measures. The values of the Figure represent results over the test datasets.

In Figure 9, it can be seen that PCA is a good competitor to RBMs for detecting temperature, although RBMs perform better than PCA in detecting temperatures below the critical temperature. Above it, the performance of both alternatives is similar. At temperatures above the critical temperature, the configurations tend to be disordered with a magnetization level close to zero. In contrast, configurations with metastates begin to exist below the critical temperature, so the PCA has a more challenging time discriminating from other configurations at higher temperatures. The PCA finds the directions of the greatest variance in the dataset and plots each configuration in its coordinates along these directions. In contrast, the RBM provides a nonlinear generalization of the PCA that transforms the high dimensionality of the system configurations into a low-dimensional code, turning the hidden layer into a feature detector of higher-order correlations of the individual activity of the spins. In this sense, it seems to us that the RBM is more flexible than a PCA in that it can transform the input into complex nonlinear representations.

## 4. Discussion and Conclusions

This work has shown that an RBM trained with configurations of a 2D Ising system at a given temperature can store enough information in the network weights to be used later as a configuration discriminator. The RBM converts the input information into a transformed vector, a simplified dimensionality representation of a raw system state. This vector can feed a simple MLP (previously trained) to recognize a system state at a specific temperature.

Why not simply use a neural network with enough hidden layers and train it as a classifier? Unlike a conventional feedforward neural network, the RBM is trained through an unsupervised process in which no information is presented to the class to which the configurations belong. Thus, in its original conception, the RBM is a model that maps the input data distribution into an alternative (ideally simplified) representation. When the RBM is trained with data for a specific temperature, say $T_{RBM}$, this representation may be sufficient to subsequently discriminate between Ising configurations at that temperature and those not. In other words, the RBM, in addition to informing us about how the configurations are distributed in a latent space simpler than the original space to which the system configurations belong, contains enough information to use this representation to train a classifier. Simple unsupervised learning techniques, such as principal component analysis (PCA) and autoencoders applied directly on the raw spin configurations of a typical Ising lattice, can identify the phase transition in the system [15,23]. The low-dimensional representations of the original data keep relevant phase information and, consequently, can be used to identify the states of interest in which the system is found.

This capability of the RBM could be helpful in frustrated systems with a wide range of ground states and roughness of the energy landscape. In these cases, there is no possibility of finding analytical solutions in advance in which one knows that under certain temperature conditions, the system could undergo a phase transition. Also, in situations where there are no singular phase transitions with abrupt changes and broken symmetry but there is a crossover region, the RBM could help to identify them. It has been shown that this is possible [37] but by using a Variational Autoencoder [38], which also achieves a dimensional data reduction in unsupervised learning. For example, with just data on the configurations of a system, one could train an RBM that takes “special” configurations and, consequently, use that RBM to detect configurations that fall into that “special” domain. To be more specific, let us think of the financial system, where we collect all the states (previously represented in a binary system) and train an RBM only on conditions of that system when it is in crisis (high volatility, for example).

Although the RBM seems useful as an alternative to creating a latent representation of an Ising system and a discriminator of configurations between different temperatures, the same cannot be said for a generator of new configurations, particularly in the ordered phase. The Ising model is characterized by a symmetry break at temperatures below the critical temperature, in which the system tends to polarize in one of the two magnetization states (+1 or −1). Under the same temperature, the distribution of the system configurations will be bimodal. This work shows that under these conditions, the RBM can capture “on average" the magnetization, correlation, energy and other measurements, but this does not imply that it can adequately reproduce system configurations. When examining the configurations generated by the RBM (at temperatures $T\leq T_{crit}$), they fail to capture the characteristic polarization of the Ising system, instead reproducing average configurations. This does not occur at temperatures $T>T_{crit}$ since there is no correlation between states in the disordered phase, and the distribution of the states tends to be offset around a magnetization around zero. This problem was initially detected by [32] by training RBMs at different temperatures but keeping the magnetization fixed at zero ($M_{0}=0$).

The RBM is not a good generator of Ising-system configurations at temperatures below the critical temperature due to the dominance of metastable states; consequently, the chains fail to mix in a reasonable amount of time [35]. This study did not solve this problem, but it may be addressed in future research by considering other types of sampling techniques that deal with data with multimodal distributions.

An actual solution to this problem, according to [39], is to predefine a concentration at a magnetization $x_{0}$. The previously trained RBM generates a sample. If it has magnetization $x_{0}$, it is accepted. If the magnetization is less than $x_{0}$, the number of spins in state −1 must be reduced, so a node *k* in state −1 is randomly selected and rebinarized according to Equation (A3). The process is repeated until the desired magnetization is achieved. The same idea applies when the sample has a magnetization greater than desired. The disadvantage of this solution is that in systems with a large number of spins, the sample-generation process can take a long time; however, it is a solution so far that manages to generate synthetic samples consistent with the actual system. Another alternative is to extend the RBM with local and shared connections to a convolutional layer [40] so that the machine can capture and preserve the spatial structure of the configurations.

I would like to point out that our study does not show the ability of the RBM to reproduce physical observables of the RBM, which is shown to be possible in several papers (as, for example, in [14,41]), but rather highlights the difficulty of correctly reproducing the bimodality observed in the Ising-model configurations at low temperatures. We have observed that once the RBM has been trained with low-temperature configurations when reproducing synthetic configurations, the average observables may agree with those measured from the data. However, when looking at the individual configurations, we see that they are not representative of those corresponding to the configuration of that temperature. We can note that the distribution of the magnetizations of the synthetic configurations fails to reproduce the bimodality of the distribution of original configurations adequately. Note that sampling feeding to the input layer contains configurations with spins predominantly with an orientation of +1 or −1 simultaneously. However, if we train considering only configurations with predominantly +1 (or −1) spins, the RBM can reproduce physically correct samples with excellent coherence of the magnetization distribution. An alternative way to overcome this problem is to alter the input configurations by imposing the constraint of leaving the predominant orientations at +1 or −1 for all the training configurations. In other words, making s become −s. This arrangement, while not altering the physical distribution of the orientations, destroys the original bimodality of the probability distribution and changes the distribution of the energies of the system as well because E(s)≠E(−s). Again, an additional constraint can be imposed on the RBM such that E(s)=E(−s) by making the visible and hidden layer biases vanish (suggested by Fernandez-de-Cossio-Diaz et al. [42]) and using the centering trick (Melchior, et al., 2016) [43]. Recently, Béreux and colleagues [44] noted the problem of the RBMs to reproduce synthetic samples in the presence of highly clustered distributions by implementing a Tethered Monte Carlo (TMC) method, a form of biased sampling to approximate the negative part of the log-likelihood gradient. This line of research could be highly relevant to expanding the data domain for unsupervised learning with energy-based models.

Although in the classification problem, the RBM performs quite well, new quantum learning models can help overcome some difficulties in generating synthetic samples that are representative of the physical system. In this sense, quantum RBMs can offer new development perspectives [45,46].

In summary, I envision that RBMs have a high potential for applicability in highly complex systems, particularly in retaining essential information in the latent parameters. The latent representation of the states into the RBM can be handy for detecting states or phases of the system, without necessarily possessing a priori knowledge of the interactions among units or the energy-functional form.

## Figures and Tables

**Table 1:** Performance measures for the MLP classifier achieved in train and test sets using hidden units of ${\mathrm{RBM}}_{T=2.3}$: area under the curve (AUC), F-score (F), accuracy (ACC), precision (PREC), sensitivity (TPR) and specificity (TNR). Values are averages over ten different MLP models with the same architecture. Values in parentheses are standard deviations.

Sample	AUC	F	ACC	PREC	TPR	TNR
Train	0.924	0.886	0.878	0.844	0.937	0.819
	(0.034)	(0.035)	(0.045)	(0.061)	(0.036)	(0.098)
Test	0.914	0.867	0.859	0.832	0.909	0.809
	(0.033)	(0.032)	(0.041)	(0.058)	(0.050)	(0.096)

**Figure A1:**
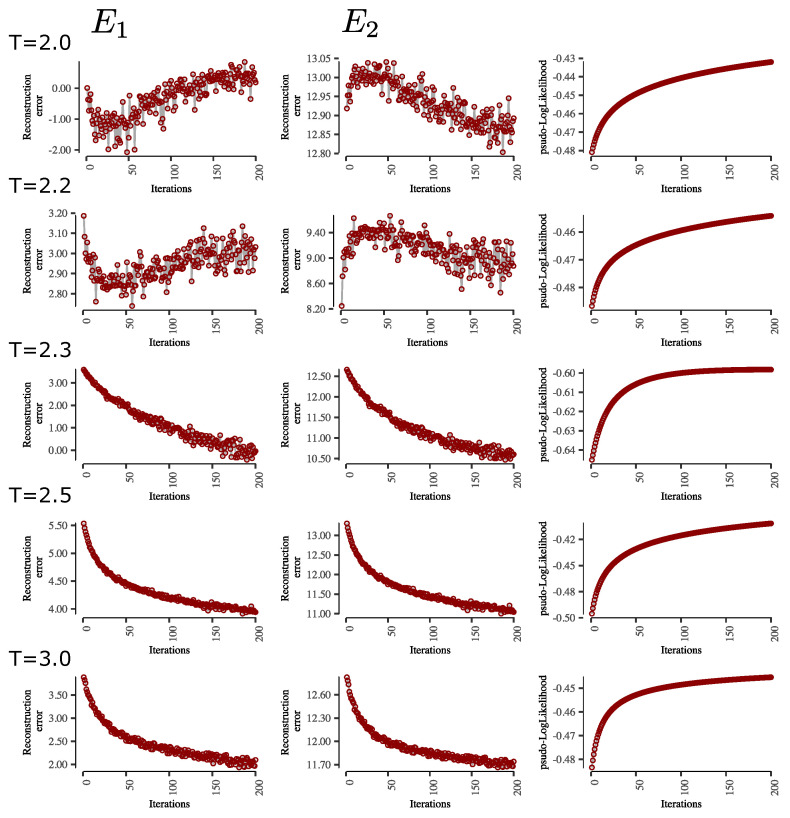
Examples of reconstruction errors through the learning process of RBM, $E_{1}$, $E_{2}$ and pseudolikelihood for different temperatures. All training using same hyperparameters: 200 iterations, nH=64 hidden units, batch size of 128, momentum = 0.8, k = 20 steps of Gibbs samplings and initial learning rate of 0.0001 with decay of 0.1.

**Figure A2:**
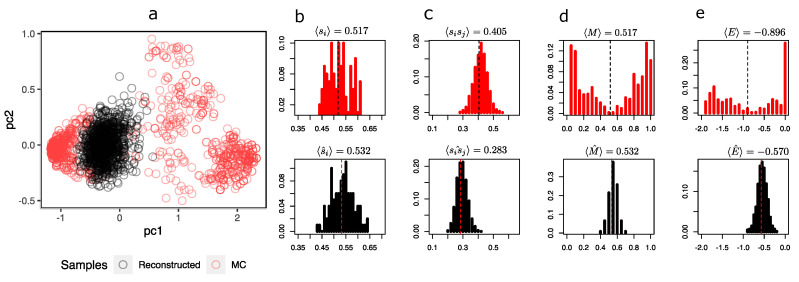
(**a**) First two principal components of 2048 reconstructed samples via Gibbs samplings using k=10 steps with ${\mathrm{RBM}}_{T=2.2}$ and 2048 MC samples at T=2.2. The explained variance with these two components was 73.9%. (**b**) Distribution of mean orientations of every spin of the lattice. (**c**) Distribution of pairwise product means between spins. (**d**) Distribution of the magnetizations of sample configurations of the Ising system. (**e**) Distribution of the system energies. Note: For all plots, black represents data from reconstructed samples and red represents data from MC samples.

**Figure A3:**
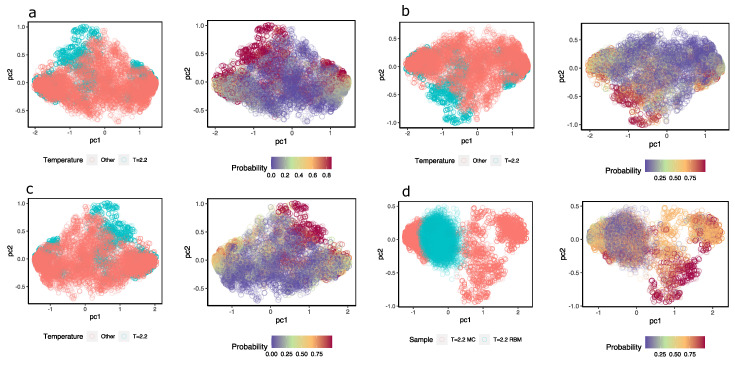
(**a**–**c**) Three exercises of configuration detection at temperature T=2.2. The figures show the first two components of the hidden values calculated with ${\mathrm{RBM}}_{T=2.2}$ in two versions: The first is colored according to the temperature of the configurations; in magenta are configurations with T=2.2 and in pink are configurations at T≠2.2. The second is colored according to the probability that y=1 is what the MLP classifier assigns to each hidden value. (**d**) The first two principal components of configurations at T=2.2; in magenta color configurations reconstructed with Gibbs samplings using k=10 iterations, in pink color configurations from Ising model samples. On the right, the configurations are the same, but they are colored by probability that the hidden units come from configurations at T=2.2.

**Figure A4:**
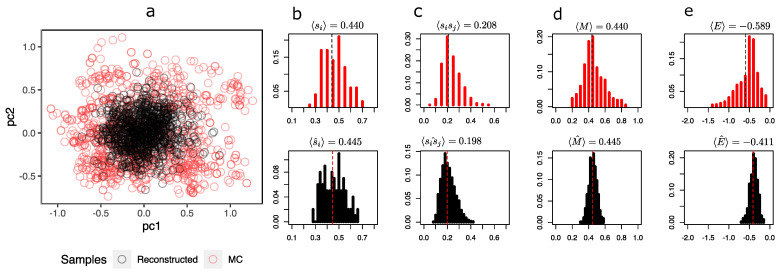
(**a**) First two principal components of 2048 reconstructed samples via Gibbs samplings with k=10 with the ${\mathrm{RBM}}_{T=3.0}$ and 2048 MC samples at T=3.0. The explained variance with these two components was 21.8%. (**b**) Distribution of mean orientations of every spin of the lattice. (**c**) Distribution of pairwise product means between spins. (**d**) Distribution of the magnetizations of sample configurations of the Ising system. (**e**) Distribution of the systems energies. Note: for all plots, black represents data from reconstructed samples and red represents data from MC samples.

**Figure A5:**
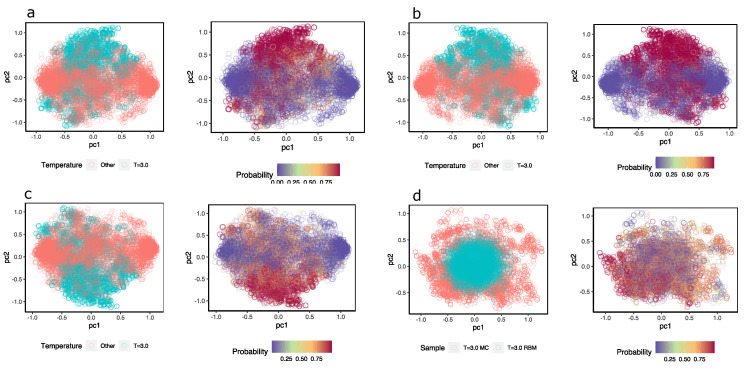
(**a**–**c**) Three exercises of configuration detection at temperature T=3.0. The figures show the first two components of the hidden values calculated with ${\mathrm{RBM}}_{T=3.0}$ in two versions: The first is colored according to the temperature of the configurations; in magenta are configurations at T=3.0 and in pink are configurations at T≠3.0. The second is colored according to the probability that y=1 is what the MLP classifier assigns to each hidden value. (**d**) The first two principal components of configurations at T=3.0; in magenta color configurations reconstructed with Gibbs samplings using k=10 iterations, in pink color configurations from Ising model samples. On the right, the configurations are the same but are colored by probability that the hidden units come from configurations at T=3.0. All computations are performed by using test samples.

## Data Availability

The simulated samples of the 2D Ising system with N=100 spins through the MC algorithm that were used in this study, are in a csv file available from the author.

## Abbreviations

The following abbreviations are used in this manuscript:

CD	Contrastive divergence
PCD	Persistent Contrastive Divergence
RBM	Restricted Boltzmann machine
MC	Monte Carlo
MCMC	Monte Carlo Markov chain
MLP	Multilayer perceptron

## Appendix A

Recall that Equation (5) indicates the probability of finding the system in a certain configuration s, which is described by the Boltzmann distribution [27]. The *restricted* condition of the Boltzmann machine means that there are no connections between neurons in the hidden layer nor between neurons in the input layer, so the hidden units are mutually independent given the value of the visible units. Likewise, the visible units are mutually independent of the hidden units. Therefore, the conditional probabilities for the visible and hidden units, respectively, can be written as

(A1) $$p\left(s|h\right)=\prod_{i=1}^{m}p\left(s_{i}|h\right)\;p\left(h|s\right)=\prod_{j=1}^{n}p\left(h_{j}|s\right)$$

where

(A2) $$p\left(h_{i}|s\right)=\sigma \left(c_{j}+\sum_{i=1}^{n}w_{ij}s_{i}\right)$$

(A3) $$p\left(s_{j}|h\right)=\sigma \left(b_{i}+\sum_{j=1}^{m}w_{ij}h_{j}\right)$$

and σ=1/(1+exp(−x)) denotes the sigmoid function.

## Appendix B

As indicated in Section 2.2, the learning consists of solving the optimization problem, where the function to minimize is the KL divergence between the probability model p(s) and the objective probability q(s) (see Equation (7)), where s is samples of the configurations drawn with Markov chain MC sampling at some temperature *T* from an Ising system. One way to monitor the learning progress is to compute the reconstructions [26] of the batches entering the input layer of both the mean pairwise products between spins $\langle s_{i}s_{j}\rangle$ and the mean orientations $\langle s_{i}\rangle$. Operationally, I take the natural logarithm of the square of the difference between the visible units in the input layer (data) and the reconstructions (model):

(A4) $$\begin{array}{cc} E_{1} & =\sum_{i\neq j}\ln[{\langle s_{i}s_{j}\rangle }_{\mathrm{data}}-{\langle s_{i}s_{j}\rangle }_{\mathrm{model}}{]}^{2}\\ E_{2} & =\sum_{\forall i}\ln[{\langle s_{i}\rangle }_{\mathrm{data}}-{\langle s_{i}\rangle }_{\mathrm{model}}{]}^{2} \end{array}$$

where $E_{1}$ and $E_{2}$ represent the similarity measure between the mean orientations and pairwise products of the Ising-system sample and the reconstruction achieved by the machine, respectively.

Also, it is possible to observe the evolution of the learning curves by using the log likelihood (see Equation (3)); however, this function becomes analytically intractable. To obtain an estimate of this quantity (a pseudolikelihood), the first sum of the log-likelihood gradient can be used to observe whether there is convergence in the process. In this case, one can use the probabilities $p(h_{i}|v^{k})$, i=1,…,N, in the Gibbs sampling algorithm for the contrastive divergence [28].

Figure A1 shows the reconstruction errors of the average orientation of the spins $E_{1}$ and of the pairwise connections between spins $E_{2}$ at different RBM-training temperatures. As can be seen, in general, the pseudolikelihood values increase at a decreasing rate, indicating the finding of an optimum in the learning process. In almost all cases, there is a rapid and consistent decrease in the reconstruction errors at the beginning and then a slower decrease. However, this is different for RBM training with Ising-system configurations in the ordered phase, i.e., when there is a clear bimodality in the magnetization of the spins. This can be explained by the fact that the RBM finds an optimal solution for the θ parameters, equivalent to an intermediate solution between the distribution of configurations with negative and positive magnetization. This explains why the spin-orientation reconstruction error $E_{1}$ drops at the beginning and starts to increase until it stabilizes.

## Appendix C

### Appendix C.1

In this appendix, I show the same exercise developed in Section 3.3 but by using an RBM trained at a temperature of T=2.2, corresponding to the system’s configurations in an ordered phase. I name this machine ${\mathrm{RBM}}_{T=2.2}$. As can be seen in Figure 1, at this temperature below the critical temperature $T_{c}$, the system tends to be in states preferentially with magnetizations very close to +1 or −1, corresponding to minimum energy, leaving large areas dominated in one of these two states.

As seen in Figure A2a, and similar to what happened with T=2.3, the RBM-generated configurations tend to be an average of the set of configurations of the MC-generated samples. Likewise, the average orientation of the spins $\langle s_{i}\rangle$ of the MC and RBM-generated samples tend to agree (see Figure A2b); however, it is clear that the magnetization of the synthetic configurations does not have the same characteristics as those of MC (see Figure A2d). The correspondence between the pairwise connections $\langle s_{i}s_{j}\rangle$ is slightly biased, indicating that the pairwise connections of the synthetic samples are undervalued relative to the MC samples.

Following the same procedure described in Section 3.5, the trained ${\mathrm{RBM}}_{T=2.2}$ is used to obtain values of hidden units that will be input to an MLP trained to detect configurations at T=2.2. The MLP parameters are the same for comparison purposes; 10 samples of independent configurations are generated by MC at different temperatures. Table A1 shows the classifier’s performance in recognizing configurations at T=2.2 for the training and test sets. All measurements are above 0.8 or very close to this value, suggesting that the RBM does a good job retaining discriminatory information between configurations in the ordered phase, similar to what occurs at the critical temperature.

**Table A1:** Performance measures for the MLP classifier achieved in training and test sets using hidden units of ${\mathrm{RBM}}_{T=2.2}$: area under the curve (AUC), F-score (F), accuracy (ACC), precision (PREC), sensitivity (TPR) and specificity (TNR). Values are averages over ten different MLP models with the same architecture. Values in parentheses are standard deviations.

Sample	AUC	F	ACC	PREC	TPR	TNR
Train	0.877	0.812	0.801	0.773	0.858	0.744
	(0.017)	(0.018)	(0.022)	(0.034)	(0.047)	(0.067)
Test	0.872	0.805	0.794	0.768	0.849	0.739
	(0.016)	(0.016)	(0.021)	(0.039)	(0.048)	(0.069)

Figure A3a–c is equivalent to Figure 8, providing a visual representation of the MLP’s ability to discriminate between system configurations that are at T=2.2 from those at T≠2.2. In Figure A3d, it is possible to observe that the configurations generated by ${\mathrm{RBM}}_{T=2.2}$ acquire a low probability of being classified as configurations at T=2.2 when in fact they should have a high probability, indicating the inability of the RBM to generate appropriate system configurations.

### Appendix C.2

As in the previous section of this Appendix, the same exercise is repeated, but this time, I am training an RBM with only T=3.0 configurations, called ${\mathrm{RBM}}_{T=3.0}$. These configurations correspond to a disordered phase characterized by very low or no spatial correlations, leaving no place to form blocks or regions with the same orientation. The mean magnetization is close to zero.

In Figure A4, unlike the previous case, it is unsurprising that the configurations are represented in the plane as a point cloud centered at zero. Similar to other temperatures, the generation of synthetic samples, while performing better, has lower variability than the MC samples. This is also observed in the distribution of the mean orientations $\langle s_{i}\rangle$ and the average magnetizations 〈M〉 of the configurations, where less variance is observed. Despite the above, the agreement regarding the means of the MC samples and those generated by the machine is satisfactory (see the means of Figure A4a–d).

When an MLP is trained (with the same procedure and architecture described in Section 3.5) to detect configurations at T=3.0 using the trained ${\mathrm{RBM}}_{T=3.0}$, we can observe that the performance is remarkable (see Table A2). All measurements are above 0.9 or very close to it. This performance is even better than in the cases for detecting configurations in the ordered phase. This is interesting because it would indicate that, in general, the RBM has the necessary information to correctly discriminate or identify a classifier configuration at a temperature equal to that of the RBM, doing slightly better when dealing with disordered phase temperatures.

**Table A2:** Performance measures for the MLP classifier achieved with training and test sets using hidden units of ${\mathrm{RBM}}_{T=3.0}$: area under the curve (AUC), F-score (F), accuracy (ACC), precision (PREC), sensitivity (TPR) and specificity (TNR). Values are averages over ten different MLP models with the same architecture. Values in parentheses are standard deviations.

Sample	AUC	F	ACC	PREC	TPR	TNR
Train	0.957	0.919	0.916	0.889	0.950	0.881
	(0.018)	(0.017)	(0.018)	(0.016)	(0.023)	(0.019)
Test	0.951	0.907	0.904	0.877	0.939	0.869
	(0.009)	(0.017)	(0.018)	(0.019)	(0.026)	(0.023)

Figure A5a–c shows a visual representation of the hidden units in their first two components and colored according to the probability that the MLP assigns them to belong or not to a configuration at T=3.0. Consistency between the probabilities and the class is observed. Configurations at T=3.0 have a high probability, and configurations at T≠3.0 are assigned a low probability. On the other hand, samples artificially generated by the RBM fail to be recognized as samples at T=3.0 (Figure A5d), indicating that the synthetic samples do not have same characteristics of natural configurations.

## Appendix D

A larger number of nH neurons in the hidden layer of the RBM allows the neural network to capture a higher level of complexity in the relationships between the nonlinear dependencies that may exist in the Ising-lattice spins. On the other hand, if the number is too low, we may limit the ability to learn such relationships by having too few latent units to capture too many complex interrelationships.

In order to see if there is an effect on the predictive ability of the RBM to discriminate configurations at different temperatures, two RBMs were trained with different numbers of neurons in the hidden layer, one with nH=160, 100 and with nH=32. Then, each of the RBMs are used to train MLPs. The training of the RBM and MLPs are carried out in the same way described in Section 2.2 and Section 3.5, respectively.

The performance indicators are shown in Table A3, Table A4 and Table A5.

**Table A3:** Performance measures for the MLP classifier achieved with training and test sets using hidden units of ${\mathrm{RBM}}_{T=2.3}$ with nH=160 hidden units: area under the curve (AUC), F-score (F), accuracy (ACC), precision (PREC), sensitivity (TPR) and specificity (TNR). Values are averages over ten different MLP models with the same architecture. Values in parentheses are standard deviations.

Sample	AUC	F	ACC	PREC	TPR	TNR
Train	0.981	0.957	0.956	0.951	0.965	0.947
	(0.044)	(0.043)	(0.044)	(0.057)	(0.056)	(0.068)
Test	0.976	0.928	0.928	0.930	0.932	0.925
	(0.023)	(0.036)	(0.036)	(0.055)	(0.064)	(0.068)

**Table A4:** Performance measures for the MLP classifier achieved with training and test sets using hidden units of ${\mathrm{RBM}}_{T=2.3}$ with nH=100 hidden units: area under the curve (AUC), F-score (F), accuracy (ACC), precision (PREC), sensitivity (TPR) and specificity (TNR). Values are averages over ten different MLP models with the same architecture. Values in parentheses are standard deviations.

Sample	AUC	F	ACC	PREC	TPR	TNR
Train	0.971	0.938	0.935	0.911	0.967	0.903
	(0.038)	(0.034)	(0.038)	(0.051)	(0.024)	(0.064)
Test	0.965	0.923	0.920	0.896	0.953	0.886
	(0.032)	(0.028)	(0.032)	(0.048)	(0.027)	(0.063)

**Table A5:** Performance measures for the MLP classifier achieved in training and test sets using hidden units of ${\mathrm{RBM}}_{T=2.3}$ with nH=32 hidden units: area under the curve (AUC), F-score (F), accuracy (ACC), precision (PREC), sensitivity (TPR) and specificity (TNR). Values are averages over ten different MLP models with the same architecture. Values in parentheses are standard deviations.

Sample	AUC	F	ACC	PREC	TPR	TNR
Train	0.882	0.827	0.815	0.779	0.885	0.745
	(0.018)	(0.015)	(0.022)	(0.035)	(0.027)	(0.061)
Test	0.873	0.816	0.804	0.773	0.869	0.739
	(0.021)	(0.014)	(0.021)	(0.043)	(0.048)	(0.078)

In all the classifier performance measures, there is an improvement when the RBM has more hidden units. With nH=100, almost all indicators exceed the 90% level of performance, while with nH=32, although equally acceptable, they are almost all above 80%. Comparing the results with those in Table 1, we also see some performance gain in having 100 hidden neurons instead of 64. However, the performance gain achieved by increasing to nH=160 hidden units is marginal with respect to the 100 neurons. Considering the cost of increasing the number of hidden units (more training time) in models with a much larger number of spins than in this study, this parameter should be selected with greater caution. These results reinforce the idea that the RBM contains relevant information in the synaptic weights to determine whether or not an input configuration comes from the temperature of the RBM.
